# Revolutionizing smartphone gyrocardiography for heart rate monitoring: overcoming clinical validation hurdles

**DOI:** 10.3389/fcvm.2023.1237043

**Published:** 2023-08-25

**Authors:** Mohamed Elgendi, Wenshan Wu, Cuntai Guan, Carlo Menon

**Affiliations:** ^1^ Biomedical and Mobile Health Technology Lab, Department of Health Sciences and Technology, ETH Zurich, Zurich, Switzerland; ^2^ School of Computer Science and Engineering, Nanyang Technological University, Singapore, Singapore

**Keywords:** smartphone gyroscope, heart rate monitoring, gyrocardiography, cardiac waveform, heart rate measurement, public health, digital health, digital medicine

## Abstract

Accurate heart rate (HR) measurement is crucial for optimal cardiac health, and while conventional methods such as electrocardiography and photoplethysmography are widely used for continuous daily monitoring, they may face practical limitations due to their dependence on external sensors and susceptibility to motion artifacts. In recent years, mechanocardiography (MCG)-based technologies, such as gyrocardiography (GCG) and seismocardiography (SCG), have emerged as promising alternatives to address these limitations. GCG has shown enhanced sensitivity and accuracy for HR detection compared to SCG, although its benefits are often overlooked in the context of the widespread use of accelerometers in HR monitoring applications. In this perspective, we aim to explore the potential and challenges of GCG, while recognizing that other technologies, including photoplethysmography and remote photoplethysmography, also have promising applications for HR monitoring. We propose a roadmap for future research to unlock the transformative capabilities of GCG for everyday heart rate monitoring.

## Introduction

The critical importance of heart rate (HR) measurements in detecting irregularities in heart rhythm patterns and cardiovascular health conditions is well established in the literature (1–7). Traditional HR measurement techniques, such as electrocardiograms (ECG) (8), demand sophisticated equipment and specialized knowledge for accurate interpretation, rendering them costly and inaccessible for long-term, everyday monitoring. The widespread adoption of smartphones in recent years (9) has given rise to an array of smartphone-based HR measurement systems employing ECG (10, 11) and photoplethysmography (PPG) (12–14). However, the reliance on smartphone-based ECG systems specialized external hardware sensors poses challenges to precise ambulatory measurements, while the accuracy of smartphone-based PPG recordings is compromised by excessive noise.

Mechanocardiography (MCG) encompasses gyrocardiography (GCG), seismocardiography (SCG), ballistocardiography (BCG), and phonocardiography (15), and increasing numbers of studies attest to its potential for cardiac performance assessment (16). Unlike other methods, MCG directly measures cardiac mechanics induced by heartbeats (17, 18). Despite their advantages, most smartphone-based HR monitoring algorithms rely on SCG signals, with few considering GCG data for HR estimation. Furthermore, published studies on the subject have been largely authored by a small group of investigators (19–24), highlighting a lack of awareness regarding GCG’s potential for HR estimation within the broader research community.

In this perspective article, we aim to analyze the potential and challenges associated with the use of GCG for HR estimation, propose future research directions, and raise awareness of its capabilities. Moreover, we introduce four evaluation metrics to standardize the evaluation process, enabling a fairer comparison between methodologies and fostering further advancements in the field.

## Exploring the potential of smartphone-based GCG for heart rate detection

Despite accelerometers being the most commonly used sensors for heart rate monitoring, recent literature suggests that due to the heart’s helical shape up to 60% of cardiac vibrational energy is contained in the gyration signal (25, 26). Furthermore, since gyroscope measurements are not affected by gravity (27), GCG signal collection is largely independent of the user’s position or posture (27); in addition proper axis selection could even result in GCG signals outperforming a combination of GCG and SCG signals for heart rate estimation (28), raising the possibility that GCG signals could supplant the currently popular SCG signals and shape the future of HR estimation.

The practicality of this application can be significantly enhanced if it can accommodate different measuring locations and postures. Most studies have only conducted HR estimation using MCG signals collected from subjects in a supine position (19–24, 28). However, vertical postures, such as standing or walking, can modulate autonomic regulation of cardiovascular function and capture different HR patterns from supine postures (29–30), owing to an increase in hydrostatic pressure in the thigh (31). Furthermore, placing the smartphone on or near the chest, though reasonable due to its proximity to the heart, could limit the application’s usefulness. In most studies, measurements required the phone to be on a bare chest or subjects to wear light clothing, restricting its usage in cold weather conditions.

These constraints may hinder the applicability of these methods in real-life situations where changes in chest pressure may lead to signal variations when subjects assume different postures or engage in physical activities. Methods capable of accurately estimating HR using a smartphone in these contexts would represent a significant advancement in the field. Such models would not only prove valuable in HR estimation but also in identifying signal patterns and characteristics under these conditions. If the models can account for the effects of posture and physical activity on MCG signals, adjusting HR estimations based on these factors to achieve accurate HR predictions becomes feasible.

While some studies have already explored HR estimation using different subject positions (e.g., standing and walking) and measuring locations (e.g., in the hand (32) and pants pocket (33)), these methods utilized either SCG signals (32, 33) or MCG signals from non-smartphone sources. Given the potential of GCG signals, further investigation into GCG signal collection from various postures and sites for HR estimation is warranted.

**Figure 1:**
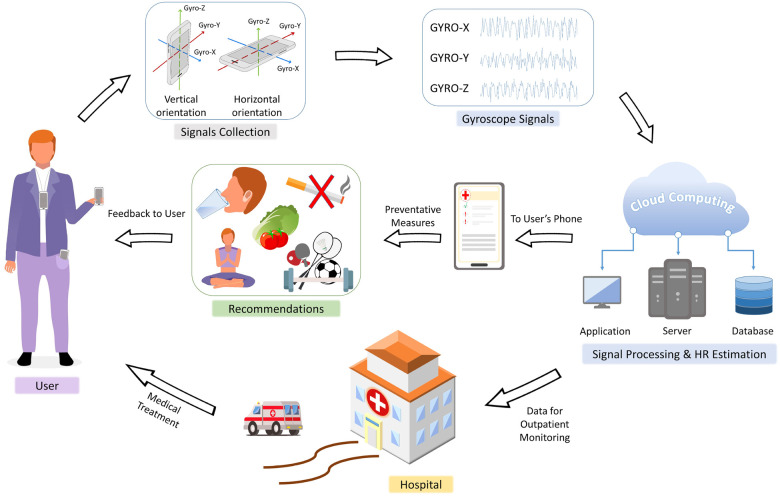
Proposed general workflow for HR monitoring using GCG signals from smartphones. The smartphones collect gyroscope data in various locations and orientations. The raw GCG signals are processed in the cloud, and computed results are sent back to the smartphone as a series of recommendations for the user. In emergency situations, an alert is sent to the hospital for a faster communication, ensuring the timely delivery of medical treatment.

Based on the literature (21, 34–39), we proposed a general workflow of using GCG signals from smartphones for HR monitoring, as shown in Figure 1. The smartphones collect GCG data from the user in various locations and orientations. The raw GCG signals are then processed (filtered, feature extraction, and then HR calculation) in the cloud. The computed HRs are sent back to the smartphone, which generates a series of recommendations as feedback to the user. In emergency situations, an alert will be sent to the hospital for faster communication to ensure that medical treatments can be delivered to the user in time.

## Obstacles encountered in smartphone GCG-based heart rate monitoring

The application of microelectromechanical systems (MEMS) vibrating structure gyroscopes (VSG) found in smartphones to heart rate monitoring is novel, and although these gyro sensors can potentially detect vital biomarkers such as blood pressure and heart rate, they were not specifically engineered for medical applications. Consequently, additional data cleaning and artifact removal steps must be implemented to achieve the necessary precision and accuracy for reliable heart rate readings.

One of the primary hurdles in employing gyro sensors for heart rate detection is the presence of motion artifacts produced by activities such as walking, running, or even typing. The orientation of the smartphone—for example, held in one’s hand versus resting on a stable surface—can also significantly impact the heart rate readings. Additionally, gyro bias error, inherent to MEMS sensors, is a significant factor in gyroscope drift error and can lead to inaccurate readings. Notably, the yaw axis is most susceptible to gyroscope drift (40). As a result, thorough time series analysis of the signals and proper axis selection are crucial for compensating for these factors and generating accurate gyroscope signals (41).

While the use of smartphone gyro sensors offers potential for heart rate detection, it is not without challenges. The accuracy and reliability of the readings can be affected by these obstacles, which must be carefully addressed when using smartphone gyroscopes for dependable heart rate monitoring.

## Wider challenges in smartphone-based heart rate monitoring

### Lack of clinical data

Before gyroscope data from smartphones can be harnessed for dependable heart rate (HR) monitoring, it is crucial to establish clinical evidence of the proposed methods’ accuracy. This demands rigorous testing and validation using a sufficiently sized and diverse participant pool, ensuring that the developed methods are unbiased and universally applicable.

Obtaining medical data, especially in emerging fields such as this, is challenging and time-consuming, as the process often requires costly specialized equipment and expert personnel. Additionally, the sensitive nature of medical data mandates strict adherence to patient privacy and data protection regulations, further complicating data collection, processing, and sharing. Despite these obstacles, concerted efforts are being made to collect and disseminate medical data for research advancement.

### Inter- and intra-subject variability

A truly effective model should account for individual variability in physiology and movement patterns. This necessitates incorporating demographic data such as race, gender, and age to create personalized models that adapt to each individual’s characteristics and behavior. Additionally, environmental factors like temperature and humidity can influence the signals HR estimation signals, as can electromagnetic interference, which can introduce noise and complicate the extraction of meaningful information.

Moreover, psychological factors, including anxiety, stress, and depression, can impact the cardiovascular system and, consequently, alter heartbeat patterns. Addressing these complexities in heart rate monitoring models ensures their adaptability and applicability across diverse scenarios and individuals, paving the way for more accurate and personalized health monitoring solutions.

#### Gender effects

The use of smartphone signal data for HR estimation involves non-invasive measurements of vibrations and rotations related to cardiomechanics using accelerometers and gyroscopes placed on the body surface near the chest. However, anatomical differences between men and women can affect the transmission of mechanical vibrations generated by the heart to the surface of the chest, leading to different directions and amplitudes of the MCG signals. Men tend to have larger and broader chests (42), while women tend to have smaller hearts, which can affect the amplitude and timing of the signals resulting from heartbeats. Differences in the shape of the heart and body fat distribution between genders (43) can also alter the mechanical vibrations transmitted to the chest. Wrist-worn wearables for HR estimation have been found to produce higher device errors in males (44), underscoring the importance of gender-specific physiological differences and the need for a balanced gender ratio in subject selection for smartphone-based HR validation studies.

#### Obesity

Obesity is a well-known cardiovascular risk factor that has been linked to an increased likelihood of heart rhythm disorders, which can have a significant impact on heart rate patterns (45–47). Anatomical fat distribution can indirectly affect the waveforms produced by the heart, as it can alter the position and orientation of the heart itself. This, in turn, can impact the mechanical forces acting on the chest wall and the signals measured by medical equipment. For example, excess abdominal fat may push the heart upwards and backwards, altering the signal’s morphology and amplitude. Additionally, differences in metabolism associated with different fat deposition patterns can also influence the function of the cardiovascular system, leading to changes in cardiac output that can influence the signal.

Although the body mass index (BMI) is widely used as an indicator of relative obesity, it is calculated from height and body weight and does not capture the body fat distribution (48). Therefore, it is generally regarded as a weak indicator of obesity. More effective anthropometric indicators such as chest circumference, waist-hip ratio, waist circumference, hip circumference, and waist stature ratio have been proposed as alternative measures of obesity.

#### Age distribution

As individuals age, the ability of cardiac muscle cells to divide and regenerate declines, which can lead to a deterioration of cardiac function and an increased risk of heart disease. Aging also causes anatomical and physiological changes within the cardiovascular system that can affect the mechanical forces acting on the chest wall (49). The heart muscle can become stiffer and thicker, especially the left ventricular wall, which can impair the heart’s ability to fill with blood and contract effectively. Additionally, the heart may increase in size (50), leading to changes in its shape and position within the chest. These changes in cardiac muscle stiffness, thickness, and size can alter the transmission of mechanical vibrations from the heart to the chest wall, leading to changes in the morphology and amplitude of the signal. Furthermore, the accumulation of lipofuscin, a pigment that results from the buildup of waste products within cells, can impair the function of cardiac muscle cells. The number of pacemaker cells in the heart also decreases with age, which can affect the heart’s ability to regulate its own rhythm (51). The alterations in the number and function of pacemaker cells can lead to changes in the timing and frequency of heartbeats, resulting in an irregular rhythm. As in obesity, aging may also lead to changes in total and regional fat distribution, with increased fat deposition often seen in internal organs, including the heart (52), further impacting the signals obtained from the heart. Overall, the interplay between these factors, as well as other comorbidities, can lead to changes in the MCG signals obtained from older adults, potentially requiring adjustments in their interpretation.

#### Comorbidity

Altered transmission of mechanical signals from the heart to the chest wall can occur with comorbidities such as hypertension and diabetes, which can cause changes in arterial stiffness (53), or respiratory diseases like chronic obstructive pulmonary disease (COPD), which can affect lung mechanics (54). Inflammatory conditions like rheumatoid arthritis and systemic lupus erythematosus can change joint and muscle mechanics (55, 56), which can affect the position of the chest wall relative to the heart. Other comorbidities that impact the autonomic nervous system, such as heart failure and atrial fibrillation, can lead to changes in heart rate variability and other measures of cardiac autonomic function and to variations in the amplitude and timing of MCG signals (57, 58). Thus, it is important to consider the presence of various comorbidities when interpreting MCG signals, as they can introduce confounding factors affecting the accuracy and reliability of measurements and downstream HR predictions.

### Performance evaluation metrics

Many studies have used smartphone MCG data, including GCG signals, to derive HR, but without proper evaluation metrics for HR estimation; however, performance evaluation metrics are essential to evaluate HR estimation methods for accuracy and precision, especially when health and safety decisions are involved. Standardized and consistent metrics for evaluating HR estimation performance can enable objective compare different HR estimation methods, improve reliability, identify effective and efficient methods, and highlight areas for improvement.

There are two aspects to having unified and consistent metrics for evaluating HR estimation performance: a standardized gold standard and evaluation metrics. Some studies have used suboptimal reference methods like HR derived from pulse oximeters or PPG signals, which themselves have varying degrees of error. This can introduce unnecessary measurement errors and potentially undermine findings. Therefore, the gold standard of ECG should ideally be used as the reference signal for HR estimation.

This article proposes four metrics for standardizing the evaluation of HR estimation performance: mean absolute errors (MAE), root mean squared errors (RMSE), Pearson correlation coefficient (r), and equivalence testing. The MAE measures general measurement error, the RMSE detects large prediction errors, the r metric measures the overall statistical relationship between estimated and reference values, and equivalence testing investigates agreements between estimated and reference measurements. A 95% equivalence testing (α=0.05) would be typically used.

Equivalence testing is preferred over the standard two-sample t-test because it confirms statistical equivalence and requires acceptance criteria to be known a priori (59–61). However, it requires the criterion to be based on either empirical evidence or domain knowledge. Researchers could follow previous studies (62, 63) that pre-defined an equivalence zone of ±10% of the reference mean, and perform a statistical test to determine whether the 90% confidence interval from the estimations falls within the equivalence zone with 95% precision (α=0.05).

The lack of standards for evaluating and reporting accuracy reduces transparency and accountability of the method, which could lead to contradictory results, lack of generalizability, false findings, and reproducibility issues. Utilizing a unified and consistent set of standards can avoid these problems, improve the clinical utility of findings in this field, and facilitate objective comparisons of different HR estimation methods.

## Conclusion

HR estimation using GCG signals from smartphones is a new and promising field that has gained attention in recent years. However, there are still relatively few studies dedicated to this purpose, which could be attributed to a lack of awareness about the potential of gyroscopes for HR detection. Our work aims to increase awareness of this field and highlight the potential of GCG signals for HR estimation. We also presented some of the challenges that need to be addressed and proposed a gold standard and four evaluation metrics for performance evaluation. By considering these factors and utilizing standardized performance evaluation metrics, comparing results across studies and establishing standard protocols for data analysis can become easier, advancing this field and potentially leading to more robust downstream classification applications.

## Data Availability

The original contributions presented in the study are included in the article/Supplementary Material, further inquiries can be directed to the corresponding authors.
